# Exploring the dynamics of fear of missing out in primary school students: examining predictors and outcomes through latent transition analysis

**DOI:** 10.1186/s40359-026-03975-y

**Published:** 2026-01-09

**Authors:** Qi Dai, Yong Hu, Baojuan Ye, Liuyan Ren

**Affiliations:** 1 School of Psychology, Jiangxi Normal University, 99 Ziyang Avenue, Nanchang, 330022 China; 2 School of Foreign Languages, Jiangxi Normal University, 99 Ziyang Avenue, Nanchang, 330022 China; 3 School of Educational Science, Henan Vocational University of Science and Technology, Zhoukou, China; 4 School of Psychology, Center of Mental Health Education and Research, Jiangxi Normal University, 99 Ziyang Avenue, Nanchang, 330022 China

**Keywords:** Fear of missing out, Individual-centered approach, Random intercept latent transition analysis

## Abstract

**Background:**

The pervasive nature of social media has given rise to the phenomenon of fear of missing out (FoMO), which poses significant challenges to the mental health of children. By drawing on an individual-centered approach, this study aims to provide a nuanced understanding of FoMO.

**Methods:**

We delved into the evolving nature of FoMO patterns among 541 Chinese primary school students within a one-year interval. Employing Latent Profile Analysis (LPA) and Random Intercept Latent Transition Analysis (RI-LTA), we uncovered three distinctive profiles of FoMO: Social Sentinels, Worry Warriors and Untroubled Buddies. To predict transitions between these profiles, we examined anxiety and mindfulness. We also investigated the relationship between two related outcomes and latent transitions.

**Results:**

The results revealed that the Untroubled Buddies profile was relatively stable, while the Social Sentinels and Worry Warriors profiles showed more frequent transitions over time. Regarding the antecedents, our findings demonstrated that primary school students with high anxiety were more likely to be categorized under the Social Sentinels and Worry Warriors profiles, increasing their risk of transitioning from the Untroubled Buddies profile to the other two profiles over time. Regarding the associations with outcomes, significant differences emerged among the three profiles in terms of emotional and behavioral problems as well as depression at three measurement points.

**Conclusions:**

Identifying different FoMO profiles among primary school students and understanding the nature of transitions between these profiles have implications for developing targeted interventions to mitigate FoMO risks among primary school students, as well as preventing individuals from transitioning into profiles associated with higher risks of emotional and behavioral problems and depression.

## Introduction

The concept of Fear of Missing Out (FoMO) is an intriguing topic that has gained significant attention in recent years, particularly with the exacerbation of excessive social media engagement and the proliferation of social media content. It has been found that individuals pay much attention to what their peers are doing, experiencing, or possessing something rewarding through the visibility of social information provided by social media [1]. Przybylski et al. (2013) define FoMO as a pervasive apprehension that others might be having rewarding experiences from which one is absent [2]. This phenomenon is closely linked to feelings of exclusion from desired experiences or an intense desire to maintain constant connectivity with others’ activities. However, this preoccupation with missing out may distract individuals from appreciating what they already have and thus may lead to detrimental effects on behavior, physical and mental health. According to a survey conducted by Roberts (2019), approximately 75% of young adults struggle with FoMO and 15.2% of respondents indicated that FoMO affects their daily life [3]. Indeed, there is a wealth of empirical evidence suggesting that higher levels of FoMO appear to be associated with low general mood [4], reduced life satisfaction [5], insomnia [6], increased likelihood of binge-drinking [7], and problematic smartphone use [8]. Hence, it is a critical area to further investigate the manifestation of FoMO characteristics and their developmental stability or changes prior to the onset of detrimental psychological outcomes.

Recent assessments on FoMO have revealed inconsistent profile patterns across different individuals. A study conducted on primary school students demonstrated a significant prevalence of FoMO among them, primarily characterized by the feelings of unease when unable to participate in social events [9]. In a separate study of 1125 young adults, latent profile analysis distinguished five distinct subtypes of FoMO [10]. Similarly, a study with 920 undergraduates has identified four FoMO subtypes [11]. Despite two studies exploring the heterogeneity of FoMO among young adults, empirical research investigating its diverse nature among primary school students remains limited. As proposed by Kelly (1955), Personal Construct Theory suggests that each person has a unique set of constructs that help them make sense of their experiences [12]. FoMO, being an affective and cognitive phenomenon rooted in the perceived disparity between one’s current and possible experiences [13], is influenced by these individual constructs. A three-wave longitudinal study using RI-CLPM reveals that 72% of the variance in the three measures of FoMO over time can be attributed to the differences between persons. This underscores the significance of considering individual differences when examining this phenomenon and emphasizes its potential as a long-term characteristic influencing individuals’ behaviors and experiences [14]. Accordingly, conducting longitudinal studies through an individual-centered approach to investigate the patterns and their transformation in FoMO holds paramount significance for gaining a more profound understanding of the underlying developmental mechanisms.

With a notable increase in internet penetration among primary school students, increased internet usage raises the possibility of suffering from FoMO [15]. According to Vasey’s influential model of worry (1993), children, particularly those in the later stages of childhood, experience an increase in the capacity, complexity, and elaboration of worry, enabling them to anticipate future events and elaborate on threatening possibilities [16]. This enhanced capability implies that primary school students start contemplating a wider array of potential situations, including positive experiences they might miss out on. This awareness not only enriches their social and emotional understanding but also brings about a new layer of complexity, which is the fear of missing out on these valuable opportunities. Moreover, studies have found that when engaging social media, primary school children are more inclined to focus on maintaining social relationships [17]. Their FoMO tends to stem from concerns about belongingness in relationships, rather than the broad self-evaluation driven by social comparisons, which is more common in adults. This distinction highlights a unique aspect of FoMO in primary school children, suggesting FoMO likely varies across different developmental stages [18]. However, the latent class-based classification of FoMO for participants was mainly focused on young adults, and cross-sectional data cannot infer the evolving nature of FoMO patterns. The random intercept latent transition analysis (RI-LTA) approach is a longitudinal extension of LPA, which goes further to estimate the incidence of transitions from one subtype to another, allowing for the estimation of stability and transition patterns among subtypes [19]. Therefore, the current study seeks to investigate the distinct patterns and transformations of FoMO among primary school students by using RI-LTA.

As previously noted, little research has been done to identify factors involved in the formation and transformations of FoMO profiles. Given the impact of FoMO on primary school students, understanding the risk and protective factors that play a role in transformations between profiles of FoMO is an important endeavor. According to Gupta and Sharma (2021), FoMO may start with distorted thinking related to the sense of fear of being left out of a rewarding experience [20]. Building on Hirsch and Matthews’ cognitive model (2012) as a framework, appraising individual pathological worry and awareness of negative intrusions (e.g., imagining that others might have more fun or are more included than themselves) are likely to have an impact on FoMO [21]. To find out whether these factors were also associated with specific profile patterns and its transition, an additional objective of the present study was to examine two related antecedents that have been identified in the previous literature: anxiety and mindfulness. On the one hand, previous studies have confirmed that anxiety disorders significantly predict FoMO [22]. Anxiety is characterized by a heightened state of arousal and distress, often manifesting as apprehension and worry [23]. This mental state is marked by a sense of dread and physical discomfort, such as restlessness and an accelerated heart rate [24]. Based on the empirical research, it is illustrated that the average prevalence of anxiety among primary school students in China was 12.3% between 2010 and 2020, showing a worsening trend with the development of the years [25]. Moreover, studies have demonstrated a medium-to-large relationship between FoMO and anxiety [26]. As previously conceptualized, FoMO is a dysfunctional cognitive response to negative affective states such as anxiety [27].

On the other hand, high mindfulness could make individuals less engrossed by negative feelings and thoughts associated with FoMO by focusing on what is happening right now, rather than what might be happening elsewhere [28]. Mindfulness is usually defined as paying attention to one’s present experience, referring to a state of being aware of ongoing physical, cognitive, and psychological experiences in a non-judgmental, accepting, and self-empathetic manner [29]. According to the reperceiving mode of mindfulness [30], individuals with higher mindfulness can disidentify from the contents of consciousness (i.e., one’s thoughts) and view their moment-by-moment experience with greater clarity and objectivity. This interrupts the automatic processing of present experiences (e.g., missing out on rewarding experiences) and the various reactions that come with it (e.g., FoMO), thereby reducing the degree of cognitive automation and the intensity of emotional responses. Therefore, this may have implications for classification and temporal movement of profiles at the individual level. In this regard, focusing on potential antecedents such as anxiety and mindfulness that sustain or weaken FoMO may help clarify the role of these antecedents in the patterns of FoMO and in the longitudinal transitions of these patterns over time. By doing so, it could inform the design and implementation of effective interventions targeting specific groups of primary school students based on their FoMO profiles.

Moreover, it was well established that FoMO was associated with negative psychological outcomes in many cases, such as emotional and behavioral problems [31, 32]. Because the persistent need to check social media interferes with sleep and productivity, research has shown that FoMO contributes to sleep deprivation and impaired focus [33]. People with FoMO frequently feel more socially isolated and alone despite having high levels of online connectivity, which can have long-term effects on their emotional and behavioral problems [34]. Beyond its impact on general emotional and behavioral problems, FoMO has also been shown to have a unique relationship with depression. Recent research indicates that FoMO is associated with depression [35]. As they pay more attention to negative information, people with higher levels of FoMO are more likely to suffer from depressive symptoms [36]. Given its widespread occurrence and profound long-term effects on an individual’s development, investigation into the association between depression and FoMO is warranted. However, do all individuals with FoMO exhibit the same levels of emotional and behavioral problems and depression? Several studies have relied on variable-centered methods, which focus primarily on overall variability and fail to capture the outcome of specific patterns of FoMO. By adopting an individual-centered approach, our study facilitates the exploration of how individuals may transition between these groups over time and the unique characteristics of different groups in terms of emotional and behavioral problems and depression.

### The present study

As prior research has indicated, the FoMO is a dynamic construct, and exhibits distinct characteristic types [37]. A longitudinal design is needed to capture these dynamic changes and understand their evolution over time, particularly in late childhood. Random intercept latent transition analysis (RI-LTA) is particularly well-suited for this purpose. As a longitudinal analytic approach, RI-LTA identifies how individuals shift between different latent states of FoMO by modeling transitions between latent classes over time. By utilizing LPA and RI-LTA to study FoMO, our study would uncover the complexity of FoMO, track changes over time, and explore the impact of anxiety and mindfulness on the dynamics of FoMO characteristics, as well as the differing patterns of these transitions in relation to mental health and depression. By developing a more nuanced understanding of the factors influencing FoMO and its consequences, we can identify how mindfulness and anxiety contribute to either the stabilization of FoMO profiles or facilitate shifts between them. Importantly, this research highlights the differences in outcomes related to mental health and depression, illustrating how variations in FoMO profiles can impact psychological well-being. By revealing these distinctions, we provide valuable insights into the potential effectiveness of mindfulness-based and anxiety-targeted interventions aimed at reducing FoMO and promoting better mental health outcomes.

Thus, the present exploratory study aimed to (1) Identify the distinct qualitative groups of FoMO among primary school students, and examine how they evolve over time. (2) Examine the associations between mindfulness and anxiety with a specific profile and the transition from one profile at one time point to another profile at the subsequent point. (3) Explore whether distinct patterns of FoMO indicate varying levels of emotional and behavioral problems and depression.

## Method

### Participants

A cluster sampling method was employed to recruit participants from two public primary schools located in Nanchang, Jiangxi Province, in central China. Both schools were randomly selected from a government-provided list of institutions compiled by local education authorities, with approximately 600 students each representing various socioeconomic backgrounds. The student body includes both urban residents and rural migrant children, making the sample more representative of the diverse population in the region. To express our appreciation while preserving the voluntary character of participation, we gave students small gifts in the form of stationery when they completed the survey. Data collection occurred across three longitudinal waves spaced at six-month intervals. In June 2023 (T1), December 2023 (T2), and June 2024 (T3), 619, 565, and 579 primary school students participated in this survey, respectively.

After excluding unqualified participants, a total of 541 valid participants were finally collected in all three waves, primarily due to school transfers, extended student leave, incomplete or patterned responses (e.g., repetitive answers), unverified identities, and administrative absences during one or more survey waves. Previous simulation study for the RI-LTA method conducted by Muthén and Asparouhov (2022) found that a sample size of *N* = 500 is acceptable for analyses involving three time points, with *N* = 1,000 yielding even better outcomes. Thus, we suppose our sample size is adequate for providing reliable findings [19]. These participants consisted of 285 boys (52.7%) and 256 girls (47.3%), aged 9–12 years (*M* = 10.47, *SD* = 0.80). The grade distribution included 264 students in Grade 4 (48.8%) and 277 students in Grade 5 (51.2%). To test whether the excluded participants were missing at random, we confirmed that no significant differences were identified between the missing participants and the follow-up participants at baseline for the main variables (*t*_FoMO−T1_ = 1.38, *t*_Mindfulness−T1_ = -0.13, *t*_Anxiety−T1_ = 1.87, *t*_Emotional and behavioral problems−T1_ = 1.64, *t*_Depression−T1_ = 1.91, all *p* > 0.05).

### Measures

#### Fear of missing out (FoMO)

FoMO was measured by using the Chinese version of the Fear of Missing Out scale [38]. This instrument assesses FoMO as a trait, or a general tendency to be mindful in daily life. The scale consists of 10 items, including “I fear others have more rewarding experiences than me.”. Each item was assessed on a 5-point Likert scale from 1 (Not at all true of me) to 5 (Extremely true of me). Higher average scores indicate higher levels of FoMO. This scale showed good reliability and validity in a Chinese sample [39]. Cronbach’s *α* for the present study was 0.82 at T1, 0.83 at T2 and 0.86 at T3.

#### Mindfulness

The Child and Adolescent Mindfulness Measure (CAMM) was used to assess mindfulness of children [40]. Children answered 10 items on a five-point scale (0 = never, 4 = always). Item responses were averaged to form a scale score, where higher scores indicated a lower level of mindfulness. The Child and Adolescent Mindfulness Measure showed good reliability and validity in studies involving Chinese children and adolescents aged 9–13 years as subjects [41]. Cronbach’s *α* for the present study was 0.89 at T1, 0.92 at T2 and 0.92 at T3.

#### Anxiety

The Generalized Anxiety Disorder Questionnaire (GAD-7) was utilized for the assessment of anxiety symptoms [42]. The GAD-7 is a 7-item self-report scale developed to assess the defining symptoms of anxiety. Items are rated on a 4-point Likert-type scale (0 = not at all to 3 = nearly every day). GAD-7 items describe some of the most salient diagnostic features of anxiety (i.e., feeling nervous, anxious, or on edge and worrying too much about different things). Scores on this scale range from 0 to 21 with higher scores indicating more severe anxiety symptoms. This scale showed good reliability and validity in a Chinese sample [43]. Cronbach’s *α* for the present study was 0.90 at T1, 0.93 at T2 and 0.95 at T3.

#### Emotional and behavioral problems

The Strengths and Difficulties Questionnaire (SDQ) is a brief, 25-item, measure of emotional and behavioral problems, which is widely recognized as a reliable instrument for evaluating emotional and behavioral problems in children and young people aged 4–16 years [44]. A Likert-type response format with three options (0 = “Not true”, 1 = “Somewhat true”, 2 = “Certainly true”) is used. Both positively and negatively phrased items are included, but all positively worded items in SDQ have been reversed. A total difficulties score was generated by summing the scores for the four sub-scales of emotion, conduct, hyperactivity-inattention and peer problems (20 items). Higher scores represent more problems, except the pro-social behaviours sub-scale, where the resultant score can range from 0 to 40. Cronbach’s *α* for the present study was 0.78 at T1, 0.80 at T2 and 0.83 at T3.

#### Depression

The Patient Health Questionnaire 9 (PHQ-9) was used to assess depression [45]. The PHQ-9, a shorter version of the complete PHQ, is a 9-item self-report scale designed to assess symptoms of depression. Each of the nine items can be scored from 0 (not at all) to 3 (nearly every day), and the total scale score ranges from 0 to 27. Higher scores indicate more symptoms of depression. Besides, the PHQ-9 was used in both the patients’ and the general population groups [46]. Cronbach’s *α* for the present study was 0.86 at T1, 0.89 at T2 and 0.90 at T3.

### Statistical analysis

All data were analyzed in SPSS 26.0 and Mplus 8.3. To utilize all possible data, we use the FIML in Mplus for missing data. Previous studies have suggested that this is the best practice method for handling missing data in structural equation models, which are unbiased and more efficient than the other methods [47].

Firstly, the descriptive and correlation analyses were performed using SPSS 26.0. A measurement invariance test was conducted in Mplus 8.3 to examine whether the constructs of main variables remained invariant over three measurement points [48].

Secondly, we utilized Mplus 8.3 to conduct latent profile analysis to explore the subtypes of children’s fear of missing out, employing 500 random sets of start values and 100 iterations. The LPA models with 2- to 5-class solutions were evaluated over three measurement points, and they were compared based on fit indices. The optimal model had lower AIC, BIC, and aBIC, and significant LMR and BLRT p-values, along with a higher entropy value [49]. Furthermore, we computed an RI-LTA to document the transition probabilities from profiles estimated at T1 to profiles estimated at T2 and from profiles estimated at T2 to profiles estimated at T3. In contrast to the single-level approach taken by traditional LTA, RI-LTA is a multilevel model in which time is nested within subjects [19]. This approach allows latent transitions to be interpreted as intra-individual changes over time by differentiating between-subject heterogeneity, captured by a random intercept, from within-subject variation. Following Muthén and Asparouhov’s (2022) suggestion, the RI-LTA model was fitted and compared with the traditional LTA model using BIC, where smaller values denote improved model performance [19].

Thirdly, multinomial logistic regressions were used to investigate the influence of demographic factors, children’s mindfulness and anxiety on the different FoMO subgroups based on the results of the LPA at Time 1. Odds Ratio (OR) results were utilized in multinomial logistic regression to determine the likelihood of children belonging to the target group under the influence of the covariates compared to the reference group. Then, distal outcome analyses were conducted using the Bolck-Croon-Hagenaars (BCH; [50]) method to compare the mean levels of emotional and behavioral problems and depression outcomes across the FoMO profiles based on the results of the LPA.

Finally, RI-LTA with covariates was performed to assess the impact of covariates on transitions. Specifically, we tested the relations between covariates at Time 1 and profile memberships at Time 2, and between covariates at Time 2 and profile memberships at Time 3. Then, to verify whether emotional and behavioral problems and depression were affected by membership in the various latent profiles, students’ emotional and behavioral problems and depression at three time points were added to the RI-LTA model as additional indicators of the profiles at their respective time points (i.e., emotional and behavioral problems and depression at Time 1 was included as an indicator of the profiles estimated at Time 1). To test for mean level differences between the latent status, we used the MODEL TEST command of Mplus, which provides an omnibus Wald chi-square test of mean differences across the profiles [51].

## Results

### Descriptive statistics

Table 1 shows the descriptive statistics and correlations among the main variables at three time points. All main variables measured were significantly and positively correlated. The levels of FoMO observed in our study are align with those found in prior research conducted among primary school students, and further support the risk of FoMO among primary school students.

**Table 1:** Bivariate correlations between main variables at three measurement points

Variables	*M *(*SD*)	1	2	3	4	5	6	7	8	9	10	11	12	13	14	15
1.FoMO(T1)	1.97(0.79)	1														
2.FoMO (T2)	1.93(0.79)	0.43	1													
3.FoMO (T3)	1.95(0.84)	0.40	0.47	1												
4.Mindfulness(T1)	0.91(0.91)	0.46	0.37	0.32	1											
5.Mindfulness(T2)	0.90(0.97)	0.34	0.45	0.38	0.58	1										
6.Mindfulness(T3)	0.93(0.99)	0.30	0.39	0.54	0.50	0.65	1									
7.Anxiety(T1)	4.23(4.98)	0.39	0.44	0.32	0.54	0.42	0.40	1								
8.Anxiety(T2)	4.03(5.42)	0.32	0.48	0.40	0.46	0.60	0.53	0.55	1							
9.Anxiety(T3)	3.99(5.69)	0.21	0.42	0.49	0.40	0.49	0.70	0.49	0.61	1						
10.EBP(T1)	13.92(5.89)	0.54	0.49	0.38	0.51	0.46	0.40	0.58	0.53	0.44	1					
11.EBP(T2)	13.46(6.53)	0.36	0.50	0.41	0.45	0.50	0.44	0.46	0.60	0.49	0.55	1				
12.EBP(T3)	13.70(6.31)	0.26	0.41	0.46	0.35	0.45	0.57	0.39	0.49	0.62	0.44	0.48	1			
13.Depression(T1)	5.22(5.59)	0.34	0.37	0.27	0.51	0.41	0.37	0.79	0.46	0.45	0.55	0.39	0.38	1		
14.Depression(T2)	4.73(5.65)	0.27	0.45	0.39	0.46	0.60	0.50	0.56	0.83	0.57	0.50	0.58	0.48	0.53	1	
15.Depression(T3)	4.51(5.77)	0.22	0.38	0.45	0.43	0.50	0.65	0.52	0.61	0.84	0.47	0.51	0.60	0.53	0.62	1

*EBP* Emotional and behavioral problems

All correlation coefficients are at the level of *p* < 0.001

### Measurement invariance

We examined the measurement invariance of main variables at three-time points, due to the necessity that measured constructs must be equivalent between time points in the longitudinal study [52]. In line with previous studies, the change of CFI (ΔCFI) was a stable indicator for model comparison due to being less influenced by sample sizes and model parameters [53]. For instance, if ΔCFI ≤ 0.01, the models would be determined to be invariant. Several nested models were compared to assess invariance across all three measurement time points. As shown in Table 2, all models fit the data adequately. Measurement invariance analysis indicated that the criterion of scalar invariance was met, suggesting that the same latent construct was measured by each indicator variable with equivalent factor loadings over time. Thus, a longitudinal model could be carried out in the next step.

**Table 2:** Model fitting results of measurement invariance for study variables

	*χ*^2^(*df*)	*p*	CFI	TLI	RMSEA	SRMR	ΔCFI
FoMO							
M0	857.916(354)	< 0.001	0.939	0.925	0.051	0.053	
M1	884.694(370)	< 0.001	0.937	0.926	0.051	0.055	-0.002
M2	920.414(386)	< 0.001	0.935	0.927	0.051	0.055	-0.002
Mindfulness							
M0	1050.724(360)	< 0.001	0.926	0.910	0.060	0.036	
M1	1078.870(376)	< 0.001	0.924	0.912	0.059	0.039	-0.002
M2	1104.791(392)	< 0.001	0.923	0.915	0.058	0.039	-0.001
Anxiety							
M0	459.922(165)	< 0.001	0.964	0.955	0.057	0.030	
M1	475.636(177)	< 0.001	0.964	0.957	0.056	0.032	0.000
M2	488.398(189)	< 0.001	0.964	0.960	0.054	0.033	0.000
EBP							
M0	6704.633(1584)	< 0.001	0.927	0.918	0.077	0.078	
M1	6156.477(1616)	< 0.001	0.935	0.929	0.072	0.081	-0.008
M2	6243.218(1648)	< 0.001	0.934	0.929	0.072	0.081	-0.001
Depression							
M0	837.239(294)	< 0.001	0.924	0.910	0.058	0.048	
M1	867.814(310)	< 0.001	0.922	0.912	0.058	0.051	-0.002
M2	925.542(326)	< 0.001	0.916	0.910	0.058	0.052	-0.006

M0 configural invariance model, M1 factor loading invariance model, M2 indicator intercept invariance model

*EBP* Emotional and behavioral problems

### Latent profile analysis of FoMO

**Fig. 1:**
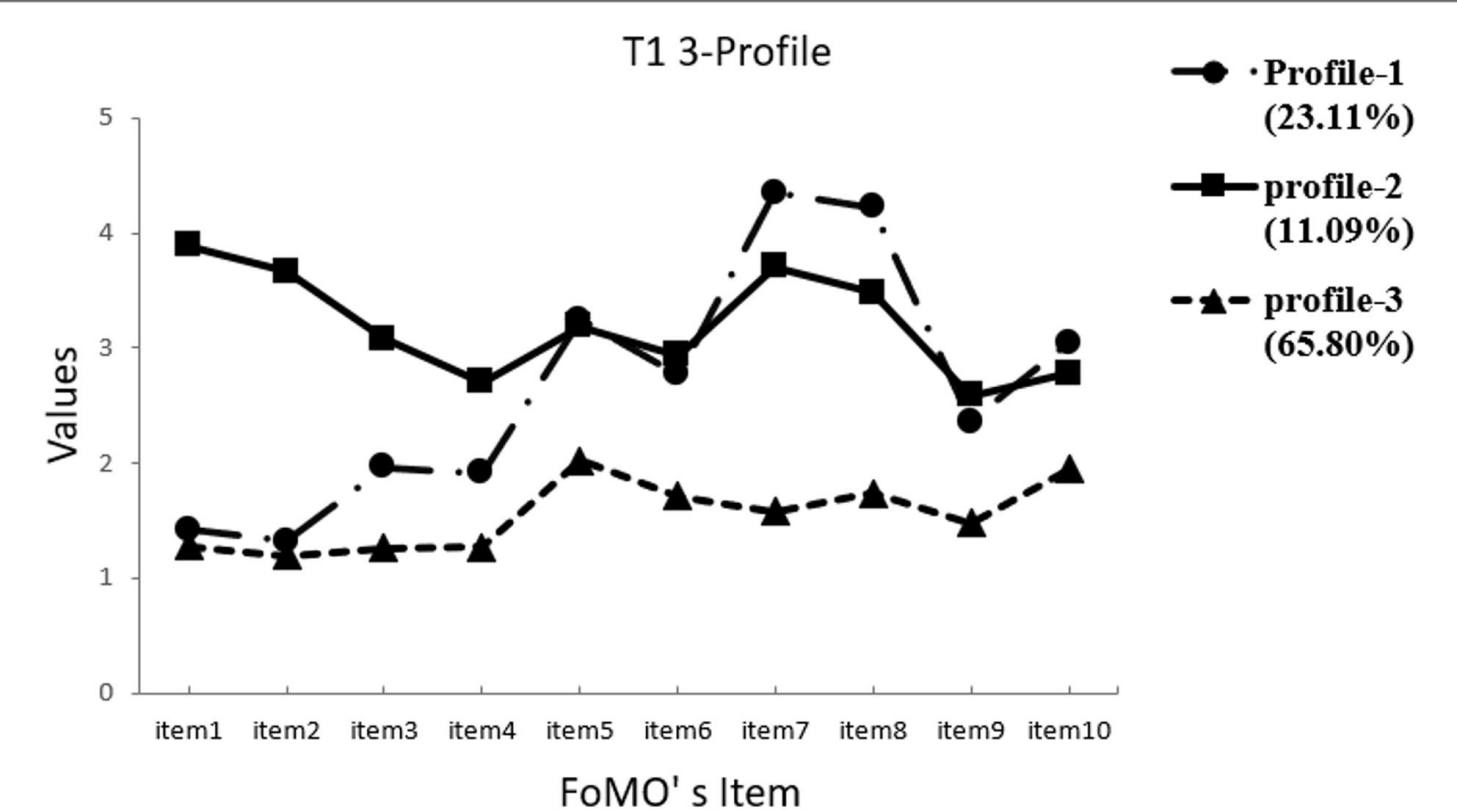
Time 1 Latent profiles of participants based on FoMO values. Profile-1 = Social Sentinels subgroup, showed higher scores on items 5, 7, 8, 10 of FoMO; Profile-2 = Worry Warriors subgroup, showed higher scores on items 1, 2, 3, and 4 of FoMO; Profiles-3 = Untroubled Buddies subgroup, showed lowest scores in all items of FoMO.

**Fig. 2:**
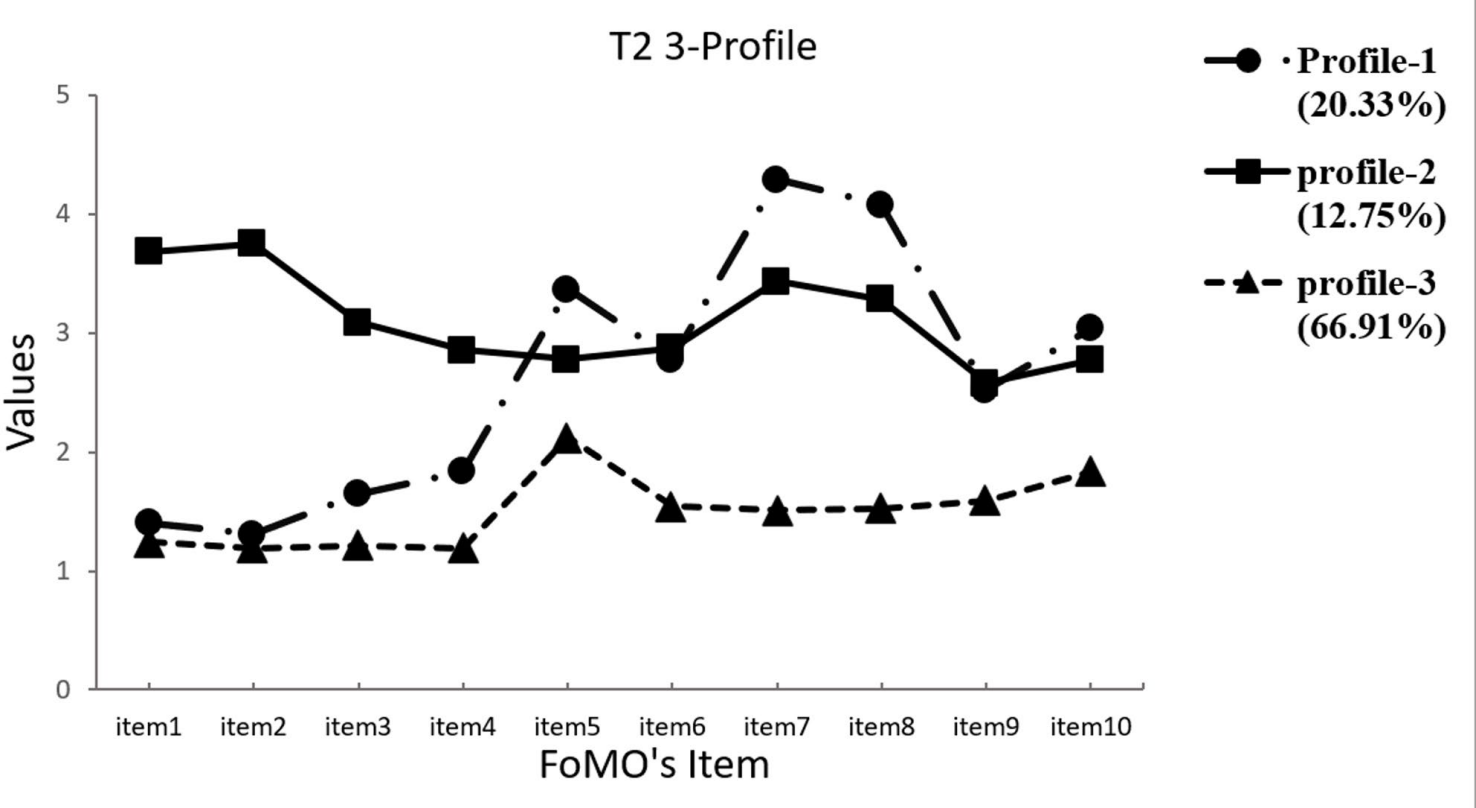
Time 2 Latent profiles of participants based on FoMO values

**Fig. 3:**
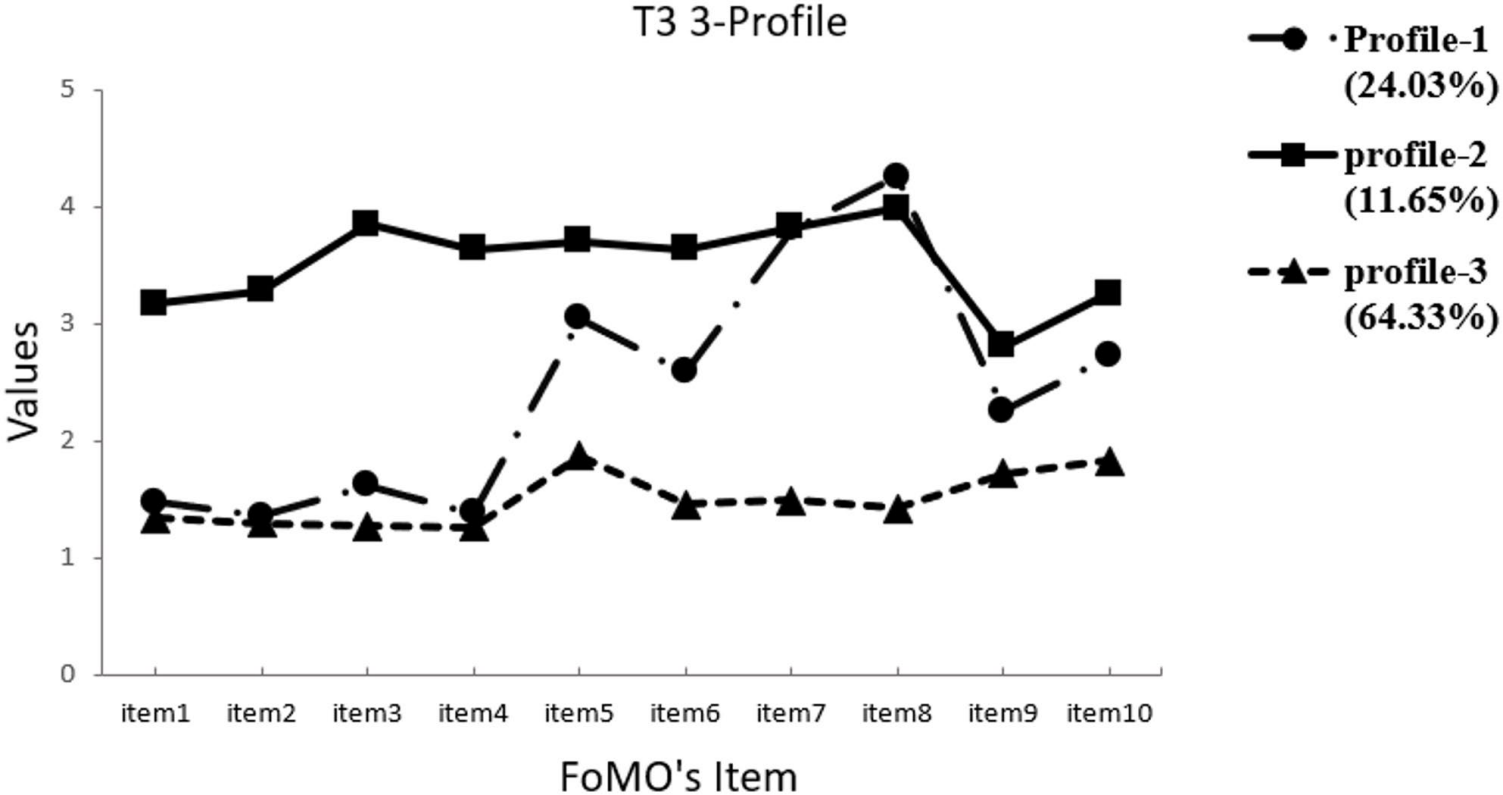
Time 3 Latent profiles of participants based on FoMO values

Referring to the suggestions of Nylund et al. (2007), the two-profile model was used as a reference point [54]. The profile models (2–5) were tested to determine the optimal model (Table 3). In our analysis, the results indicate that the LMR-LRT significance for the 4-profile and 5-profile solutions does not meet the required standards. Additionally, the AIC, BIC, and aBIC values for the 2-profile solution are higher than those for the 3-profile solution. Therefore, a model with three profiles at each time point was considered the best fitting solution. The scores distribution of the latent profile of fear of missing out on all items at three time points are presented in Figs. 1 and 2, and 3.

**Table 3:** Fit indices for four models using LPA (*N* = 541)

Profile	Free parameters	AIC	BIC	aBIC	LMR-LRT(*p*)	BLRT(*p*)	Entropy
*Time 1*							
2-profile	31	16696.388	16829.484	16731.078	< 0.001	< 0.001	0.918
3-profile	**42**	**16133.893**	**16314.217**	**16180.893**	**0.020**	**< 0.001**	**0.936**
4-profile	53	15833.393	16060.945	15892.703	0.115	< 0.001	0.954
5-profile	64	15548.666	15823.445	15620.286	0.226	< 0.001	0.946
*Time 2*							
2-profile	31	16060.426	16193.522	16095.117	0.210	< 0.001	0.968
3-profile	**42**	**15288.833**	**15469.157**	**15335.834**	**< 0.001**	**< 0.001**	**0.953**
4-profile	53	14857.201	15084.753	14916.511	0.618	< 0.001	0.946
5-profile^a^	—	—	—	—	—	—	—
*Time 3*							
2-profile	31	16313.184	16446.280	16347.875	< 0.001	< 0.001	0.964
3-profile	**42**	**15622.772**	**15803.096**	**15669.773**	**0.005**	**< 0.001**	**0.944**
4-profile	53	15211.494	15439.045	15270.804	0.457	< 0.001	0.938
5-profile	64	14912.776	15187.555	14984.396	0.249	< 0.001	0.944

Numbers in bold indicate the optimal solution; ^a^ 5-profile solution in Time 2 has encountered issues with local maxima; (1) Lower AIC, BIC, and aBIC values indicate better model fit; (2) LMR-LRT(*p*) = p-value for Lo Mendell-Rubin adjusted likelihood ratio test for K vs. K-1 profiles, BLRT(*p*) = p-value for Bootstrapped Likelihood Ratio Test, a statistically significant LMR-LRT or BLRT test result (*p* < 0.05) indicates that the model with k classes better fits the model than with one latent class less (i.e., k-1 classes); (3) Higher values for entropy (i.e., > 0.80) provide supporting evidence that profile classification of individuals in the model is realized with minimal uncertainty [55]

Profile 1 included 23.11% (*n* = 125) at time 1, 20.33% (*n* = 110) at time 2 and 24.03% (*n* = 130) at time 3 of the child and was labelled as “Social Sentinels”, due to their higher scores on items 5, 7, 8, and 10(i.e., item5- It is important that I understand my friends ‘‘in jokes’’; item10- When I go on vacation, I continue to keep tabs on what my friends are doing), highlighting their focus on building and maintaining positive social relationships. The children of profile 1 are motivated by a desire to stay connected with and to stay actively engaged with their friends, stay updated on social events and enjoy shared experiences. Profile 2 consisted of 11.09% (*n* = 60) at time 1, 12.75% (*n* = 69) at time 2 and 11.65% (*n* = 63) at time 3 of children who scored higher on items 1, 2, 3, and 4(i.e., item1- I fear others have more rewarding experiences than me; item4- I get anxious when I don’t know what my friends are up to), labeled as “Worry Warriors”, showing they driven by fear, anxiety, and insecurity, more likely to feel left out or worried, reflected in high emotional arousal and emotional experience. Profile 3 consisted of 65.80% (*n* = 356) at time 1, 66.91% (*n* = 362) at time 2 and 64.33% (*n* = 348) at time 3 of children who showed low fear of missing out, labelled as “Untroubled Buddies”, suggesting they are the least worried about others’ experiences or keeping up with social activities. The three subgroups were employed in subsequent analyses.

### Latent transition analysis of FoMO

Based on the LPA results, we initially tested the traditional LTA model and the RI-LTA to document the transition probabilities from profiles estimated at T1 to profiles estimated at T2 and from profiles estimated at T2 to profiles estimated at T3. The RI-LTA was chosen due to its superior model fit indices compared to the traditional LTA. Specifically, the RI-LTA demonstrated lower BIC (see Table 4) compared to the traditional LTA, indicating a better fit to the data. Additionally, both models exhibited sufficient entropy (higher than 0.8), suggesting that accurate classification of individuals has been achieved. Therefore, RI-LTA was utilized for subsequent analysis.

**Table 4:** Model fit of traditional LTA and RI-LTA

Models	AIC	BIC	aBIC	Entropy
LTA	46841.477	47159.190	46924.288	0.936
RI-LTA	45861.051	46221.698	45955.052	0.934

**Fig. 4:**
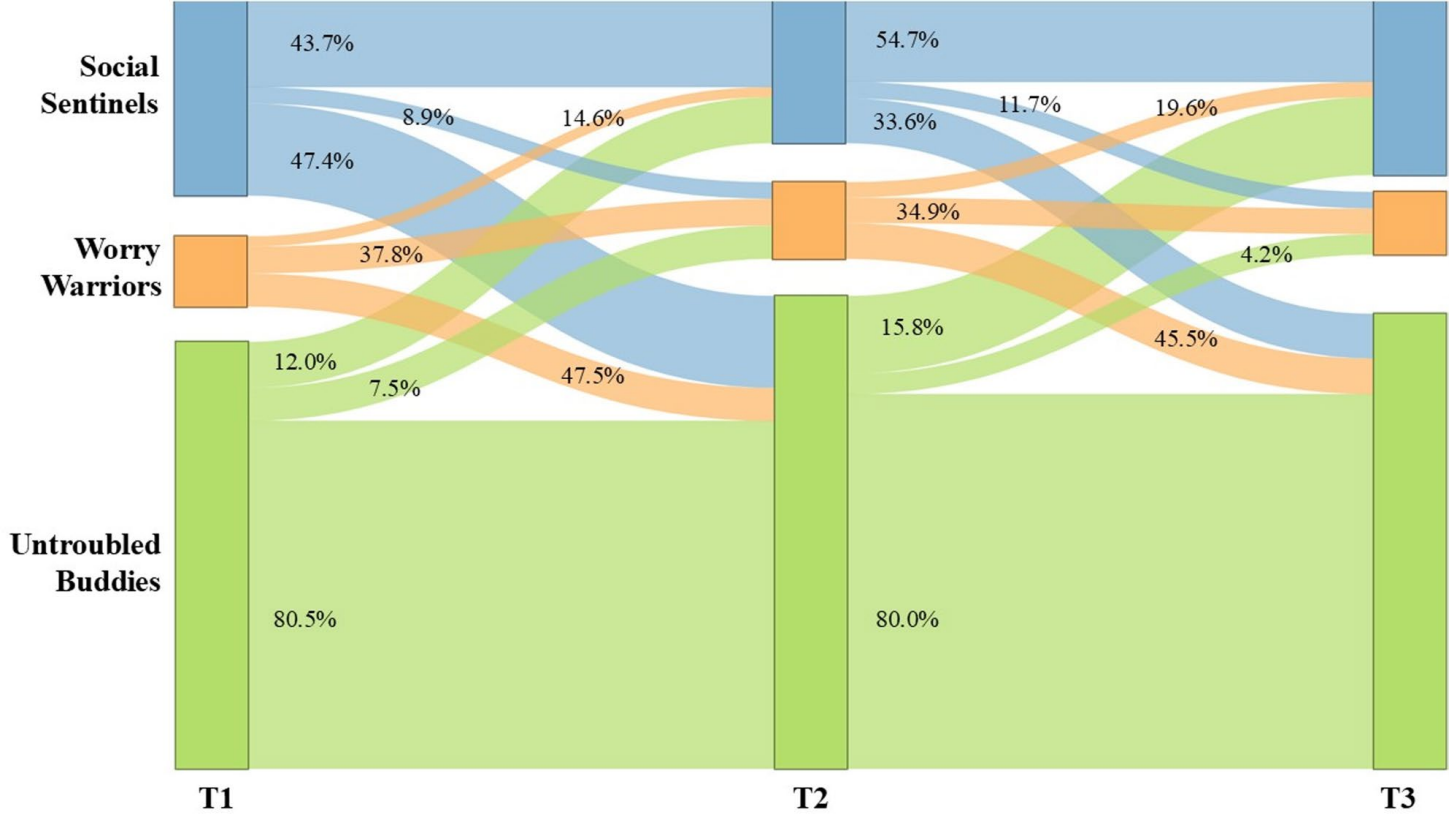
FoMO profile transitions from T1 to T2 and T2 to T3

The stability and transition probability matrices of latent procrastination profiles are presented in Table 5. Given a person’s profile membership at time t, the transition probability indicates the possibility of that individual shifting to a certain profile at time t + 1. The diagonal values represent the odds of status stability, whereas the off-diagonal values indicate the probabilities of profile transitions [56]. The most stable profiles in terms of transitions between Time 1 and Time 2 were the Untroubled Buddies profile with an 80.5% likelihood of remaining in that profile at T2, followed by a 12.0% likelihood of transitioning to Social Sentinels, and a 7.5% likelihood of transitioning to Worry Warriors at T2. Whereas children in the Social Sentinels profile had a 43.7% likelihood of remaining in the same profile, with an 8.9% likelihood of transitioning to Worry Warriors and a 47.4% likelihood of transitioning to Untroubled Buddies at T2. The Worry Warriors had a 37.8% likelihood of remaining in the same profile, with a 14.6% likelihood of transitioning to Social Sentinels and a 47.5% likelihood of transitioning to Untroubled Buddies at T2. Transitions between profiles from Time 2 to Time 3 were generally similar to those from Time 1 to Time 2. In terms of movement, all students had a greater likelihood of remaining in the same latent profile (ranging from 34.9% to 80.5%), with those in the Untroubled Buddies profile being the most likely to do so. Figure 4 provides the transition probabilities of each profile from Time 1 to Time 2 and Time 2 to Time 3 as a Sankey plot. The width of the flow represents the percentage of individuals moving between FoMO statuses.

**Table 5:** Transition probabilities for RI-LTA

	profiles	Social Sentinels	Worry Warriors	Untroubled Buddies
			T2	
T1	Social Sentinels	**0.437**	0.089	0.474
	Worry Warriors	0.146	**0.378**	0.475
	Untroubled Buddies	0.120	0.075	**0.805**
			T3	
T2	Social Sentinels	**0.547**	0.117	0.336
	Worry Warriors	0.196	**0.349**	0.455
	Untroubled Buddies	0.158	0.042	**0.800**

Note: Bold values on the diagonal represent the odds of profile stability (i.e., the probability of remaining in the same latent profile from Time 1 to Time 2 and from Time 2 to Time 3). Off-diagonal values indicate transition probabilities between different profiles

### Covariate effects

Based on the results of the LPA, we further tested the effects of covariates, including gender, age, anxiety, and mindfulness, on FoMO profile memberships, with Untroubled Buddies serving as the reference group in multinomial logistic regression models. The results of the multinomial logistic regression model are shown in Table 6. An OR value greater than 1 indicates that the covariate increases the likelihood that the individual belongs to the target group [57]. Lower mindfulness individuals were more likely to have higher FoMO than higher mindfulness individuals in Profile-1 (Social Sentinels; OR = 2.289, 95%CI [1.638, 3.199]) and Profile-2 (Worry Warriors; OR = 2.095, 95%CI [1.467, 2.990]) in reference to Profile-3 (Untroubled Buddies). Additionally, individuals with worse symptoms of anxiety were more likely to have higher FoMO in Profile-1 (Social Sentinels; OR = 1.500, 95%CI [1.133, 1.987]) and Profile-2 (Worry Warriors; OR = 2.188, 95%CI [1.601, 2.990]). The demographic variables were not significant enough to help distinguish between different FoMO subgroups.

**Table 6:** Association between demographic factors, anxiety, mindfulness and different forms of FoMO in multiple logistic regression analysis

Reference group is Untroubled Buddies (profile-3)	Social Sentinels (Profile-1)	Worry Warriors (Profile-2)
	OR (95% CI)	OR (95% CI)
Gender	0.912 (0.547–1.521)	0.708 (0.351–1.429)
T1 Age	0.942 (0.723–1.228)	1.988^*^(1.321–2.990)
T1 Anxiety	1.500^*^(1.133–1.987)	2.188^***^(1.601–2.990)
T1 Mindfulness	2.289^***^(1.638–3.199)	2.095^**^(1.467–2.990)

Gender was coded as 1 = male and 2 = female

^*^p < 0.05, ^**^p < 0.01, ^***^p < 0.001

Based on the RI-LTA results, we tested the effects of covariates on the transitions between FoMO latent status over time. The value of odds ratio (OR) is significantly greater than 1, suggesting an increased probability of transitioning to a different profile, whereas a value of odds ratio less than 1 indicates a reduced likelihood of such transitions, favoring stability within the same profile. Table 7 illustrates the impact of covariates on transitions from T1 to T2 and from T2 to T3, compared to remaining in the same profile. Age was associated with a decreased likelihood of transition from the Worry Warriors group (Profile-2) to Social Sentinels group (Profile-1) between T2 and T3 (OR = 0.136[0.028, 0.661]), suggesting that the individuals who increased in age have a greater stability in the high-risk FoMO group. Besides, the odds of staying in the Untroubled Buddies group (profile-3) versus moving to the Worry Warriors group (Profile-2) are 2.024 times higher for individuals with high anxiety compared to those with low anxiety (OR = 2.024[1.339, 3.059]) from T1 to T2. Similarly, students with higher anxiety were more likely to move from the Untroubled Buddies group (profile-3) to the Social Sentinels group (Profile-1) (OR = 1.524[1.005, 2.313], from T1 to T2; OR = 1.914[1.163, 3.150], from T2 to T3). However, mindfulness and gender showed no significant correlation with transitions between latent statuses.

**Table 7:** Odd ratios for covariates predicting transitions between latent statuses of FoMO

Predictor	Latent Status	T1→T2			T2→T3		
		C1	C2	C3	C1	C2	C3
Gender	C1	**REF**	1.025(0.212,4.956)	1.138(0.498,2.600)	**REF**	2.040(0.486,8.556)	1.093(0.411,2.907)
	C2	1.150(0.193,6.864)	**REF**	0.725(0.134,3.926)	1.179(0.167,8.318)	**REF**	0.804(0.201,3.212)
	C3	1.682(0.744,3.804)	1.130(0.419,3.043)	**REF**	1.296(0.659,2.550)	0.310(0.083,1.157)	**REF**
Age	C1	**REF**	1.329(0.684,2.582)	1.007(0.661,1.534)	**REF**	1.003(0.683,1.473)	1.060(0.743,1.511)
	C2	1.122(0.196,6.409)	**REF**	0.332(0.118,0.934)	**0.136(0.028**,**0.661)**	**REF**	1.029(0.374,2.833)
	C3	1.512(0.997,2.294)	0.574(0.309,1.068)	**REF**	1.007(0.711,1.425)	0.746(0.316,1.764)	**REF**
Anxiety	C1	**REF**	1.531(0.636,3.685)	1.175(0.737,1.873)	**REF**	1.096(0.630,1.905)	0.774(0.472,1.269)
	C2	1.030(0.359,2.952)	**REF**	0.515(0.241,1.098)	0.319(0.094,1.078)	**REF**	0.728(0.361,1.464)
	C3	**1.524(1.005**,**2.313)**	**2.024(1.339**,**3.059)**	**REF**	**1.914(1.163**,**3.150)**	1.440(0.761,2.726)	**REF**
Mindfulness	C1	**REF**	1.009(0.489,2.081)	0.606(0.366,1.003)	**REF**	1.548(0.895,2.677)	0.738(0.438,1.242)
	C2	0.931(0.353,2.456)	**REF**	0.864(0.366,2.040)	3.586(0.814,15.803)	**REF**	1.045(0.512,2.130)
	C3	1.257(0.842,1.875)	1.076(0.679,1.704)	**REF**	1.343(0.854,2.113)	1.846(0.549,6.201)	**REF**

Values marked in bold represent a significant effect of a predictor. Values in parentheses indicate 95% confidence intervals

Abbreviation: REF Reference profile

﻿C1 = Social Sentinels group, C2 = Worry Warriors group, C3 = Untroubled Buddies group

### Associations of the outcome with LPA/RI-LTA

As shown in Table 8, post hoc analysis indicated that Worry Warriors (Profile-2) were associated with the highest scores on emotional and behavioral problems, which means higher risk with emotional and behavioral problems, followed by Social Sentinels (Profile-1), with the lowest score being Untroubled Buddies (Profile-3). Similarly, there was a similar pattern in the outcome of depression. Notably, there was a significant difference between each subgroup in terms of emotional and behavioral problems and depression.

**Table 8:** Mean differences in emotional and behavioral problems and depression across three profiles and BCH results

Profiles	EBP *M*(*SE*)	Depression *M*(*SE*)
Social Sentinels (Profile-1)	16.235 (0.498)	6.330 (0.545)
Worry Warriors (Profile-2)	20.160 (1.000)	10.216 (0.974)
Untroubled Buddies (Profile-3)	12.051 (0.281)	3.993 (0.266)
Pairs(*χ*^*2*^)		
Omnibus Test	99.421^***^	47.437^***^
Profile-1 vs. 2	12.122^***^	11.895^***^
Profile-1 vs. 3	51.534^***^	14.280^***^
Profile-2 vs. 3	60.524^***^	37.733^***^

*EBP* Emotional and behavioral problems

*p* < 0.05, ^**^p < 0.01, ^***^p < 0.001

The results from the next set of analyses, in which emotional and behavioral problems and depression were separately included as additional profile indicators in RI-LTA, are reported in Table 9. For three time points, the omnibus test of mean differences was highly significant. Also noteworthy is the fact that the means and variances of emotional and behavioral problems within each profile are very similar across time points, again reflecting the stability of the profiles. A detailed examination of mean differences shows significant differences between most of the profiles. This can serve as supplementary proof that our grouping is distinct, effective, and conceptually meaningful. Notably, the Worry Warriors group (Profile-2) was more likely to be associated with a higher risk of emotional and behavioral problems and depression. Perhaps least surprising, in contrast to the non-risk group (Untroubled Buddies), the two risk groups (Social Sentinels and Worry Warriors) of FoMO exhibit higher levels of emotional and behavioral problems and depression.

**Table 9:** Within-Time comparisons of the profile groups on emotional and behavioral problems and depression

Outcome	Time points	Social Sentinels (Profile-1)	Worry Warriors (Profile-2)	Untroubled Buddies (Profile-3)	Omnibus Test
		*M*(*SE*)	*M*(*SE*)	*M*(*SE*)	*χ*^*2*^(*df*)
EBP	Time 1	15.027 (0.491)	21.293 (0.940)	12.174 (0.309)	101.219 (2) ^***^
	Time 2	15.583 (0.599)	19.388 (1.148)	11.953 (0.323)	61.507 (2) ^***^
	Time 3	14.549 (0.578)	20.345 (0.984)	12.318 (0.355)	59.274 (2) ^***^
Depression	Time 1	5.652 (0.487)	10.013 (1.120)	4.233 (0.300)	28.937 (2) ^***^
	Time 2	6.491 (0.664)	10.075 (1.054)	3.457 (0.246)	54.618 (2) ^***^
	Time 3	5.386 (0.675)	11.757 (0.892)	2.986 (0.268)	88.725 (2) ^***^

*EBP* Emotional and behavioral problems

^*^p < 0.05; ^**^p < 0.01; ^***^p < 0.001

## Discussion

With the rise of social media and constant digital connectivity, people are more exposed to the lives of others, which can exacerbate FoMO. To reduce the adverse effect of FoMO, the current study utilized a one-year longitudinal study design with a sample of primary school students. By employing individual-centered statistical methods, LPA was used to investigate the latent subgroups of students and RI-LTA was used to investigate their transformations based on the FoMO items at three time points, identified three profiles, namely Social Sentinels (Profile-1) and Worry Warriors (Profile-2) and Untroubled Buddies (Profile-3), described the stability and transitions of the profiles over time. Then, we examined the impacts of demographic factors, mindfulness, and anxiety on different FoMO subtypes using multinomial logistic regression based on LPA findings at Time 1, and included the above-mentioned covariates in RI-LTA to examine the different impacts of covariates on FoMO transition patterns. Finally, we added emotional and behavioral problems in LPA findings at Time 1, investigating the differences across different subtypes in terms of emotional and behavioral problems, as well as, adding them into RI-LTA to estimate the association between our final retained model and outcomes. Therefore, our results contribute to a deeper understanding of varying patterns of FoMO, its transformations, potential antecedents, and association with emotional and behavioral problems outcome. The following was a discussion of the key findings.

### Heterogeneity of primary school student FoMO

We conducted a latent profile analysis of FoMO among primary school students at three time points and found three latent profiles of FoMO, which provided a good fit to these data, suggesting heterogeneity among these students. The observed results can be explained by the theory of personal constructs, which suggests that individuals’ unique construct systems and interpretations of missing out on rewarding social events play a crucial role in shaping their experiences of FoMO.

The three FoMO subgroups were identified in this study. Specifically, students in the Social Sentinels group scored higher on items 5, 7, 8, and 10 of the FoMO, indicating that they were more committed to social events and had a constant need to stay connected and updated. Students in the Worry Warriors group displayed higher scores on items 1, 2, 3, and 4 of FoMO, indicating that they were worried about being left out of enjoyable activities and that they experienced more emotional reactions related to potential exclusion or a lack of information about friends’ activities. In contrast, students in the Untroubled Buddies group scored lowest on all FoMO items, indicating low FoMO, less stress related to keeping up with others’ experiences, and a greater appreciation for their own experiences and life circumstances. This finding diverges from previous studies investigating the latent subgroups of young adults, which identified FoMO as 5 or 4 types [10, 11]. We speculate that this might be due to the sample being at different age stages, or to previous studies often classifying groups according to different severity standards for FoMO, rather than capturing distinct patterns with distinct characteristics of FoMO. Therefore, the discrepancy between the current study and prior research highlights gaps in our understanding and warrants further exploration. Future research should explore the characteristics and main manifestations of FoMO with different emphases and across various age groups, thereby helping develop more targeted and effective interventions.

### The stability of primary school students’ FoMO

The current study advances our understanding of the temporal transition in profiles of FoMO across time. If the FoMO profiles indicate that the profile structure within a sample is reactive to situational cues (i.e., lack of temporal stability), it will be difficult to make meaningful recommendations. The transition patterns found in our study revealed a predominant tendency for stability rather than transition. Of which, the Untroubled Buddies profile was the most stable at the three time points. This group had the largest proportion, particularly, over 60% of the population labeled Untroubled Buddies, with a 70% probability of remaining in the same status for 12 months. One possible explanation for such high belonging rates and stability is that both external stimuli and individual internal factors, such as self-esteem and social sensitivity, may influence FoMO. Therefore, even in a highly connected world, the unique characteristics of each child can lead to varying responses to FoMO [58]. Another possible explanation is that although some children may possess the cognitive capacity to recognize potential positive experiences they might miss out on, their emotional responses may not be as strong, which diminishes the experience of FoMO. Additionally, children’s strong curiosity and tendency to be easily attracted by novel stimuli rather than prolonged fixation on events may contribute to this stability [59].

We noticed that both Social Sentinels status and Worry Warriors status had a certain probability transferred to Untroubled Buddies status (over 20%), presenting a trend of improvement. This may be partly accounted for by the child’s development [60]. On the other hand, it may also be due to children having a variety of extracurricular activities and hobbies, which not only enrich their lives but also provide more real social opportunities, enabling the establishment of more genuine and stable interpersonal relationships, meeting relationship needs, and thus reducing the feeling of fear of missing out. This may suggest that relevant intervention not only should encourage primary school students to reduce the frequency of social media use or social media monitoring to alleviate the fear of missing out [61, 62], but also needs to guide primary school students in developing a variety of activities and hobbies, participating in real-life social activities, building deeper and more meaningful connections with others, meeting their psychological needs, and enhancing autonomy and relational needs. This also requires exploration and practice in future clinical practice.

### Covariates effects on primary school students FoMO

The current longitudinal study applied latent profile analysis and latent transition analysis with covariates to explore the influence of demographic factors, anxiety, and mindfulness on FoMO among primary school students. The results showed that gender and age are not differentiating factors in determining the classification of FoMO, but showed some influence on transformation between profiles. Higher levels of anxiety and lower mindfulness predicted more severe FoMO profiles at the first measurement point. Interestingly, the movement from the Untroubled Buddies status to the Social Sentinels status or Worry Warriors status was predicted by the increases in the level of anxiety. However, mindfulness and gender showed no significant predictive effect on the transitions between latent statuses.

First, our findings of multinomial logistic regression showed that demographic factors were not important for being classified into any of the different subgroups of FoMO at Time 1. However, while age influences how an individual’s FoMO profile changes over time, it does not directly affect the individual’s underlying latent FoMO profile at a given point in time. In other words, while age itself does not necessarily lead to higher FoMO, it may persist in existing FoMO tendencies in some individuals. Age may be a risk factor for people who already experience heightened levels of FoMO, aligning with the concept that FoMO is an individual difference phenomenon.

Second, mindfulness showed a predictive effect on classification into different subgroups of FoMO but did not appear to affect transitions between latent statuses, consistent with previous variable-centered research [63]. This may be because FoMO is a multifaceted phenomenon influenced not only by individual traits, such as mindfulness, but also by external factors, such as social media usage patterns and the nature of online interactions. Thus, while mindfulness can offer protective benefits, it may not be sufficient alone to alter entrenched behaviors or thoughts associated with high FoMO, indicating the potential need for holistic interventions that combine mindfulness with other strategies.

Third, more anxiety symptoms consistently predicted a higher risk of FoMO based on the findings of both LPA and RI-LTA. This is consistent with previous research on how anxiety affects FoMO [64]. The characteristic of anxiety is persistent and excessive worry and a sense of tension [65], which may lead individuals with anxiety to be more inclined to be classified at risk of FoMO. Their sensitivity and vulnerability to specific social events create risk factors for moving from Untroubled Buddies status to the other two statuses over time. Thus, tailored interventions for FoMO must consider the individual’s anxiety to distinguish between the comorbidity of FoMO and anxiety. Otherwise, addressing symptoms without addressing the root cause may lead to relapse later. Implementing grouped intervention strategies, coupled with ongoing and specific adjustments, is crucial for reducing the risk of FoMO.

### Outcome with LPA/RI-LTA of FoMO

Results from the current study have provided further support for previous research indicating that FoMO, which stems from a desire to fit in socially and a fear of exclusion, is associated with emotional and behavioral problems and depression [66]. The results indicated significant differences in emotional and behavioral problems and depression among the different FoMO profiles at three measurement points. Specifically, individuals classified as Worry Warriors (Profile-2) exhibited the highest levels of emotional and behavioral problems and depression significantly. Social Sentinels (Profile-1) showed the second-highest levels, while Untroubled Buddies (Profile-3) displayed significantly lower values of emotional and behavioral problems and depression compared to all other groups. These results suggest that varying degrees of FoMO membership may confer different risk levels for potential emotional and behavioral problems and depression. This discovery offers a deeper understanding of the priority of intervention, allowing individuals to benefit from early personalized intervention measures if the pattern is identified and screened for. Consequently, appropriate personalized intervention measures and guidance should be implemented based on the relatively weaker stability observed within the Worry Warriors status and Social Sentinels status to facilitate their transition towards the Untroubled Buddies status. While students belonging to the Worry Warriors group do not constitute a high proportion of the overall population studied, they are at an increased risk of developing more severe psychological health problems and depression, thus necessitating early attention and targeted support strategies.

### Limitations and future direction

The limitations of this study should be acknowledged. Firstly, the reliance on self-report surveys may have inflated some relations between self-perception variables and negative subjective experiences [67]. Future research should consider incorporating additional methodological approaches (e.g., other informants, experience sampling methodology) to gain a deeper understanding of the underlying mechanisms related to FoMO. Secondly, this study was conducted with a sample of primary school students from a single province in China who shared a relatively homogeneous cultural background. This geographic restriction, along with the moderate sample size, may limit the generalizability of our conclusions. Caution should be taken when extending these findings to broader populations, including those from different regions, cultures, or clinical settings [68]. Future research should aim to replicate our study across different cultural contexts and include larger and more diverse samples to enhance the robustness and applicability of the findings. Thirdly, one significant limitation is the scope of factors examined. Although we considered several key variables, the multifaceted nature of FoMO suggests that numerous other situational and environmental factors could influence its development. For instance, we did not explore in detail the impact of specific social media use patterns, peer influence, and societal expectations. Future research should aim to address these gaps to provide a more comprehensive understanding of the factors contributing to FoMO.

## Conclusion

In summary, the current study advances our understanding of fear of missing out among primary school students by several key findings. Firstly, we adopt an individual-centered approach by using latent profile analysis and identified three distinct FoMO latent profiles. Secondly, we expanded the LPA findings by applying RI-LTA to uncover its transition patterns. Thirdly, we examined the role that anxiety and mindfulness play in shaping distinct FoMO profiles and transition patterns. Finally, we discussed the outcomes associated with FoMO profiles, illustrating that membership in a specific FoMO profile is distinctively associated with emotional and behavioral problems and depression, which would be missed if using a variable-centered approach, thereby providing new conceptual insights into FoMO research through RI-LTA. These findings highlight the importance of considering symptom treatment when experiencing FoMO and using subtypes of FoMO as a reference point for identification and improvement, to avoid unfocused intervention targets or methods that may lead to low effectiveness.

## Data Availability

The datasets used in this study are available from the corresponding author on reasonable request.
